# Ethical universality in medicine: A critical rational bioethics beyond cultural relativism

**DOI:** 10.1016/j.bas.2026.106268

**Published:** 2026-08-10

**Authors:** Nicolás Samprón, Naci Balak, Jehuda Soleman, Tiit Mathiesen, Marike Broekman, Mario Ganau

**Affiliations:** a Hospital Universitario Donostia, Spain; b Policlínica Gipuzkoa, San Sebastián, Spain; c Istanbul Medeniyet University Göztepe City Hospital, Turkey; d University Hospital of Basel, Switzerland; e University of Copenhagen, Denmark; f Leiden University Medical Center, the Netherlands; g Oxford University Hospitals NHS Foundation Trust, UK; h School of Medicine, BAU International University Batumi, Batumi, Georgia

**Keywords:** Common morality, Ethical universalism, Cultural relativism, Reflective equilibrium, Critical rational bioethics, Moral equality

## Abstract

**Introduction:**

Cultural diversity in healthcare raises a central question: are ethical obligations in medicine universal or culturally dependent? While modern medicine presupposes moral equality, cultural relativism challenges the universality of ethical standards.

**Materials and methods:**

Conceptual and historical analysis of medical ethics. The major ethical codes and dominant frameworks: cultural relativism, common morality, and reflective equilibrium were critically examined to assess how ethical norms develop and how ethical judgments are evaluated.

**Results:**

Ethical progress in medicine is better explained by the recognition and correction of error than by the convergence of cultural beliefs. Cultural relativism is incompatible with moral equality and risks undermining consistent patient protection. Existing frameworks provide limited accounts of ethical knowledge development. A problem-driven, critical model, “Critical Rational Bioethics”, is proposed, in which ethical judgments are treated as conjectures subjected to systematic scrutiny and revision.

**Discussion and conclusion:**

Ethical universalism follows from moral equality and is incompatible with cultural relativism. Ethical progress in medicine is best understood as the correction of error rather than the justification or convergence of beliefs. Critical Rational Bioethics provides a problem-driven, fallibilist framework in which ethical judgments are critically assessed and provisionally retained. This approach reconciles universal ethical obligations with context-sensitive implementation, supporting consistent patient protection in multicultural clinical settings.

## Highlights

• Ethical universality in medicine follows from moral equality.
• Cultural relativism risks unequal and inconsistent patient protection.
• Ethical progress arises through error correction, not consensus.
• Critical Rational Bioethics provides a problem-driven framework.
• Universal principles guide care with context-sensitive implementation.

## 1 Introduction

Modern medicine presents itself as a universal human practice. Its scientific foundations, epistemic standards, and clinical aims are assumed to apply equally to all human beings, regardless of cultural, religious, or social background. This claim to universality is not merely technical but moral: it presupposes that all patients possess equal moral worth and are therefore entitled to the same fundamental ethical protections (United Nations, 1948; UNESCO, 2005).

Despite this universalist aspiration, the application of ethical principles in clinical practice varies across cultural contexts. Differences in beliefs about autonomy, informed consent, truth-telling, and end-of-life decision-making have been widely documented, reflecting the influence of social, religious, and institutional factors (Macklin, 1999; Brannigan, 2000; Mathiesen et al., 2022; Inglehart et al., 2014). These observations have led to the view that ethical norms, while universal in abstraction, require contextual specification in practice.

Empirical evidence further supports the diversity and variability of moral beliefs across societies and over time. Cross-cultural research shows that attitudes toward autonomy, authority, and individual rights differ systematically between populations and evolve with social and economic change (Inglehart et al., 2014). This descriptive pluralism, however, does not by itself determine the validity of ethical norms.

In response, culturally sensitive approaches argue that ethical principles may be universal in theory but require contextual interpretation in practice. This position seeks to accommodate diversity while preserving core ethical commitments, emphasizing flexibility in interpretation and application across different clinical and cultural settings (Beauchamp and Childress, 2019; Tosam, 2020).

However, this strategy leaves a central tension unresolved. If ethical obligations vary according to cultural context, then the claim that all patients are equal in moral status is undermined. The boundary between legitimate contextual adaptation and variable ethical standards remains unclear, allowing cultural considerations to influence not only how care is delivered, but which ethical obligations are applied.

This difficulty is not resolved by dominant frameworks such as reflective equilibrium, which seeks coherence among moral judgments, principles, and background theories (Rawls, 1999; Beauchamp and Childress, 2019). While such approaches provide methods for organizing ethical reasoning, they offer limited accounts of how ethical knowledge develops or how flawed norms are critically revised rather than maintained within existing systems of belief.

This paper examines whether ethical obligations in medicine are universal and argues that ethical universalism follows from moral equality. It further proposes Critical Rational Bioethics as a problem-driven, fallibilist framework in which ethical judgments are treated as conjectures and subjected to systematic criticism. These challenges arise not only within multicultural societies but also across healthcare systems that differ substantially in their available resources, legal frameworks, and cultural traditions.

## 2 Conceptual foundations: moral equality, universality, common morality, and relativism

**Moral equality** is the principle that all human beings possess the same intrinsic moral worth and are therefore entitled to equal ethical consideration, irrespective of their cultural, social, or personal characteristics (United Nations, 1948). From this principle follows the **universality** of medical ethics: ethical obligations apply equally to all patients and cannot vary according to cultural context without undermining moral equality itself.

**Common morality** is often presented as the set of norms shared by all persons committed to morality and is taken to provide a universal foundation for bioethics (Beauchamp and Childress, 2019). However, this account assumes convergence of moral beliefs where empirical evidence shows persistent variation across societies and over time (Inglehart et al., 2014). By grounding ethical claims in widely shared norms, it risks treating prevalence as epistemically relevant without explaining why commonly held beliefs should track what is ethically correct.

In contrast, **cultural relativism** holds that ethical norms are determined by the values and practices of particular societies, denying the existence of universally valid moral standards and, in stronger forms, the possibility of objective moral truth (Westacott, n.d.; Macklin, 1999). On this view, differences in moral beliefs across cultures cannot be resolved by appeal to a common framework, and no standpoint exists from which competing ethical claims can be critically assessed. In clinical practice, this position risks permitting variable standards of care, thereby undermining the principle of moral equality.

## 3 A novel method of ethical reasoning: critical rational bioethics

Methods of ethical reasoning in medicine serve two related aims: to guide clinical judgment in particular cases and to contribute to the development of ethical knowledge over time. While clinicians require practical tools to make decisions in concrete situations, this presupposes a broader epistemic question: how do ethical judgments arise, and how can they improve? Ethical knowledge is not static but evolves as practices once considered acceptable are later recognized as ethically wrong (Strong, 2008; Deutsch, 2011). However, dominant approaches tend to focus on the organization or justification of moral beliefs, offering limited accounts of how ethical errors are identified and corrected (Popper, 1963; Beauchamp and Childress, 2019). This calls for a framework in which ethical reasoning is understood as a problem-driven process of critical revision.

**Fig. 1:**
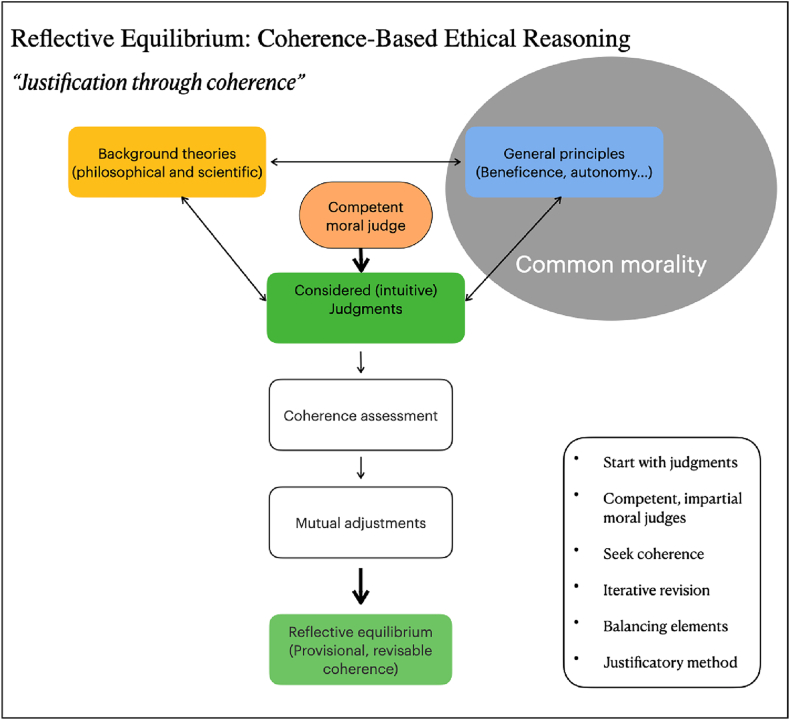
**Reflective equilibrium as a coherence-based method of ethical reasoning.** Reflective equilibrium seeks coherence among considered judgments, general principles, and background theories through a process of iterative mutual adjustment (Rawls, 1999; Beauchamp and Childress, 2019). Considered judgments are typically grounded in the perspectives of competent and impartial moral agents, whose intuitions serve as epistemically relevant starting points. Inconsistencies among elements are reduced through a process of balancing and revision until a stable and coherent configuration is achieved. The resulting equilibrium remains provisional and open to further revision, and is presented as a justificatory framework for the evaluation of moral judgments.

**Reflective equilibrium** (Fig. 1), originally developed by Rawls and later applied to bioethics by Beauchamp and Childress, is a method of moral reasoning aimed at achieving coherence among different elements of ethical thought (Rawls, 1999; Childress, 2007; Arras, 2009). It begins from “considered judgments”, i. e. moral intuitions about particular cases formed by competent moral agents under conditions intended to minimize bias. These are then brought into relation with more general principles and relevant background theories. When inconsistencies arise, these elements are mutually adjusted until a coherent and stable alignment is achieved (Rawls, 1999; Beauchamp and Childress, 2019). In this sense, reflective equilibrium is presented as a justificatory method in which coherence among judgments, principles, and theories provides epistemic support. Although the resulting equilibrium remains provisional and open to revision, the process primarily operates within existing systems of belief.

**Fig. 2:**
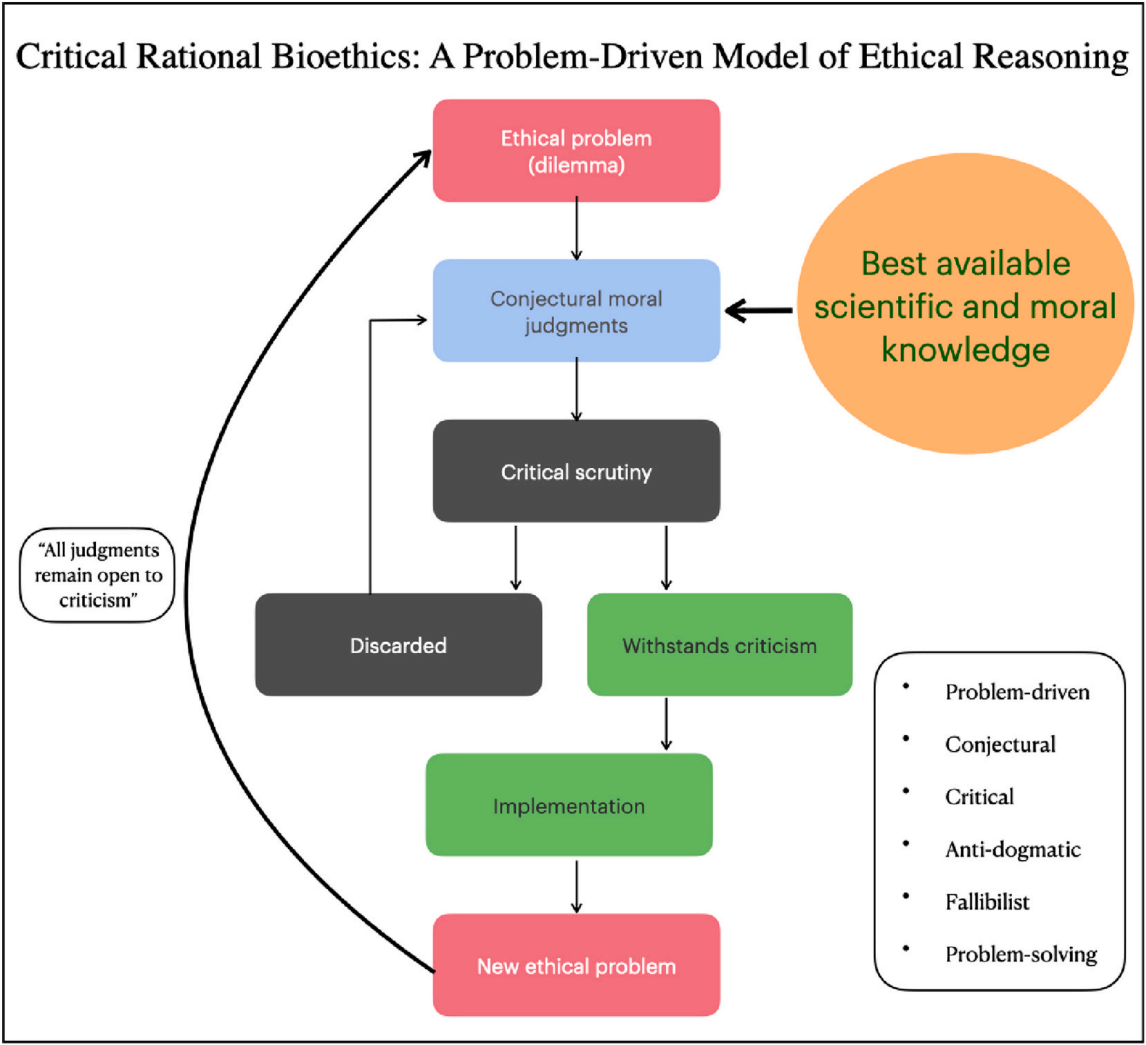
**Critical Rational Bioethics as a problem-driven model of ethical reasoning.** Ethical reasoning begins with the identification of a problem or dilemma, which gives rise to tentative moral judgments understood as conjectures. These conjectures are informed by the best available scientific and ethical knowledge and are subjected to critical scrutiny and attempts at refutation (Popper, 1963; Deutsch, 2011). Inadequate judgments are rejected, while those that withstand criticism are provisionally retained and applied in practice. The outcomes of implementation generate new problems, sustaining an iterative process of ethical knowledge development through error correction. No ethical judgment is final; all remain open to further criticism.

We propose a method of ethical reasoning and knowledge generation termed **Critical Rational Bioethics** (Fig. 2), explicitly inspired by Popperian epistemology, in which knowledge develops through problems, conjectures, and attempts at refutation (Popper, 1963, 1994). While this approach shares with reflective equilibrium the view that ethical reasoning is iterative and that judgments may require revision, it differs in its starting point, criterion of evaluation, and epistemological commitments (Table 1). Rather than beginning from the considered judgments of competent moral agents, it assumes fallible agents and starts from ethical problems—situations in which expectations fail, principles conflict, or patient protection is at risk. These problems generate tentative moral judgments understood as conjectures, informed by the best available ethical knowledge, including ethical principles, philosophical accounts, and relevant scientific and clinical understanding, which are themselves open to critical revision. These conjectures are then subjected to systematic criticism and attempts at refutation. Unlike coherence-based approaches, this process does not aim to align judgments with existing beliefs, but to expose their weaknesses and eliminate error. Ethical progress, on this view, arises not from coherence but from the critical detection and correction of mistakes (Popper, 1963; Deutsch, 2011). The outcome is a provisional judgment that has withstood sustained critical scrutiny and remains open to further revision.

**Table 1:** Comparison of coherence-based and problem-driven models of ethical reasoning. Reflective equilibrium is presented as a coherence-based approach grounded in the considered judgments of competent and impartial moral agents, and their mutual adjustment with principles and background theories (Rawls, 1999; Beauchamp and Childress, 2019). Critical Rational Bioethics (CRB), by contrast, treats ethical reasoning as a problem-driven and fallibilist process in which moral judgments are understood as conjectures and subjected to systematic criticism and refutation (Popper, 1963; Deutsch, 2011). The comparison highlights differences in starting point, epistemic orientation, method, and the mechanism of ethical progress.

Two models of ethical reasoning: coherence-based justification vs problem-driven error correction		
Feature	Reflective Equilibrium	Critical Rational Bioethics
**Starting point**	Considered judgments	Ethical problems (dilemmas)
**Epistemic basis**	Competent, impartial moral judges	Fallible ethical agents
**Nature of inputs**	Intuitions and principles	Conjectures (tentative solutions)
**Methods**	Coherence and balancing	Critical scrutiny and refutation
**Goal**	Coherent equilibrium	Error detection and elimination
**Outcome**	Stable, coherent set of beliefs	Provisional solutions that survive criticism
**Role of revision**	Mutual adjustment for coherence	Continuous criticism and error correction
**View of progress**	Increasing coherence	Error reduction (approximation to truth)
**Status of knowledge**	Justified beliefs	Fallible revisable conjectural
**Epistemic orientation**	Judgement-dependent (intersubjective)	Problem-orientated (objective, fallibilist)
**Logical constraint**	Allows coherence among conflicting intuitions	Eliminates contradictions through criticism
**Practical implication**	Stabilising existing beliefs systems	Correction of harmful or inadequate practices
**Role of authority**	Implicit (competent judges)	None (no authority-based grounding)

### 3.1 Clinical vignette 1: Cultural diversity and patient autonomy

#### 3.1.1 Problem

A neurosurgeon in a public hospital evaluates a 25-year-old woman with progressive visual loss due to a hypothalamic tumor. The patient, a recent immigrant, is accompanied by relatives and communicates through a translation device. During the consultation, a male relative who speaks the clinician's language insists that all information be directed to him rather than to the patient, without ensuring that she understands the discussion, and refuses the proposed surgical treatment on her behalf. The clinician faces the ethical problem of respecting cultural dynamics while safeguarding the patient's right to receive information and participate in decisions concerning her own care.

#### 3.1.2 Conjectures

Several possible courses of action are considered. One is to defer entirely to the relative's wishes and communicate exclusively through him. A second is to insist on direct communication with the patient despite the family's objections. A third is to postpone decision-making until effective communication with the patient can be ensured.

#### 3.1.3 Criticism

Each conjecture is critically examined in light of the physician's ethical obligations. Exclusive communication through the relative risks denying the patient meaningful access to information and undermining her autonomy. Conversely, disregarding the family's role without considering the patient's own wishes may fail to respect her cultural values and personal preferences. Postponing decision-making may be appropriate under certain circumstances but risks delaying treatment if effective communication can already be established. Rather than seeking coherence among competing moral intuitions, CRB evaluates these alternatives by identifying potential errors and determining which course of action best protects the patient's moral interests.

#### 3.1.4 Provisional resolution

The clinician ensures that the patient is given the opportunity to receive information directly, using the available translation resources, and to ask questions about her condition and treatment. Once adequately informed, the patient voluntarily chooses to delegate communication to her relative. This decision is accepted as an autonomous expression of her preferences rather than imposed by others. The duty to inform is thereby fulfilled without forcing decision-making, and the process remains open through continued dialogue and follow-up.

This vignette illustrates how cultural diversity influences the implementation of ethical obligations without modifying the obligations themselves. Within Critical Rational Bioethics, respect for moral equality, patient autonomy, and professional responsibility remains universal, whereas culturally sensitive communication represents a context-dependent strategy for implementing those universal principles.

### 3.2 Clinical vignette 2: universal ethical obligations in a resource-limited healthcare setting

#### 3.2.1 Problem

A young man is admitted after a motor vehicle accident with an unstable thoracolumbar fracture associated with an incomplete spinal cord injury. According to current evidence and accepted standards of care, the preferred treatment is urgent decompression, anatomical reduction, and posterior internal fixation. However, spinal instrumentation is unavailable at the treating hospital, and neither the patient nor his family can afford to purchase the necessary implants in the acute setting. Consequently, definitive stabilization cannot be performed within the recommended timeframe.

#### 3.2.2 Conjectures

The treating team considers two possible courses of action. The first is to refrain from surgical intervention because the optimal treatment cannot be completed according to accepted standards. The second is to perform the best treatment currently feasible by carrying out decompression and anatomical reduction, while preparing the patient for definitive stabilization should spinal instrumentation become available at a later stage.

#### 3.2.3 Criticism

The first conjecture preserves adherence to the ideal standard of care but leaves the patient without any active treatment despite the availability of potentially beneficial interventions. The second conjecture attempts to maximize patient benefit under unavoidable material constraints but introduces uncertainties, as decompression without definitive stabilization may increase instability or expose the patient to additional complications. Neither option fully satisfies the ideal standard of care, requiring critical evaluation of which course of action best fulfils the physician's ethical obligations under the existing circumstances.

#### 3.2.4 Provisional resolution

After critical discussion, the surgical team performs decompression and anatomical reduction while preparing the operative field for later instrumentation. The patient is managed with strict bed rest and external immobilization until definitive stabilization becomes feasible. Several weeks later, following financial support raised by the patient's family and local community, spinal instrumentation becomes available and definitive fixation is successfully completed.

This vignette illustrates that resource limitations modify the implementation of ethical obligations rather than the ethical obligations themselves. Within the framework of Critical Rational Bioethics, the physician's duty to act in the patient's best interests, while respecting dignity, beneficence, and professional responsibility, remains unchanged. What varies is how these universal obligations can be fulfilled under severe material constraints. Accordingly, CRB does not distinguish between an ethics for resource-rich settings and another for resource-limited settings, but between universal ethical principles and their context-dependent implementation.

## 4 Evolution of ethical knowledge in medicine: evidence of progress through error correction

The historical development of medical ethical codes provides not only a record of changing norms but evidence that ethical progress often emerges in response to moral failure. Major advances in bioethics have followed the recognition of ethical violations, prompting critical reassessment and reform. The formulation of the Nuremberg Code, the Declaration of Helsinki, and the Belmont Report, for example, arose in response to documented abuses and introduced progressively stronger protections for research participants and patients (Annas and Grodin, 1992; National Commission for the Protection of Human Subjects of Biomedical and Behavioral Research, 1979; Broekman, 2019).

As summarized in Table 2, early ethical frameworks were predominantly paternalistic, with little or no recognition of patient autonomy. Over time, autonomy emerged as a safeguard against coercion and abuse and was progressively consolidated as a central principle of medical ethics. Contemporary frameworks explicitly prioritize the rights, safety, and well-being of the individual over scientific or societal interests and reject the view that cultural or institutional considerations can override fundamental ethical obligations (UNESCO, 2005; World Medical Association, 2024).

**Table 2:** **Historical evolution of autonomy in medical ethics.** Major ethical codes are presented to illustrate the progressive emergence and consolidation of patient autonomy. Changes are interpreted as responses to identified ethical failures, supporting a model of ethical development driven by problem recognition and error correction rather than cultural variation or consensus.

Evolution of the Concept of Autonomy in Medical Ethical Codes for clinical practice and research				
Code/document	Year	Ethical model	Role of autonomy	Key ethical contribution
**Hippocratic oath**	500-400 BCE	Paternalistic	Absent	Establishes medicine as a moral practice (beneficence, non-maleficence, confidentiality)
**AMA code of ethics**	1847	Strong paternalism	Explicitly absent/patient obedience	Formalizes physician authority and duty-based ethics
**Nuremberg code**	1947	Protective/anti-abuse	Introduced (essential consent)	First explicit requirement of voluntary informed consent
**Declaration of Geneva**	1948	Professional duty-based	Absent	Reaffirms physician duties, dignity, and non-discrimination
**Helsinki declaration (original version)**	1964	Transicional	Emerging (consent required)	Formalizes research ethics, risk–benefit assessment, consent
**Belmont declaration**	1979	Principlism	Central (respect for persons)	Establishes autonomy as a foundational ethical principle
**UNESCO Declaration on Bioethics and Human Rights**	2005	Universalist/rights-based	Explicit and protected	Links bioethics to human rights; limits cultural relativism
**WMA International Code of Medical Ethics**	2022	Mature professional ethics	Core principle	Affirms patient decision-making and independence from external constraints
**Helsinki declaration (revised version)**	2024	Fully developed/rights-priority	Central and overriding	Establishes primacy of individual over science and society

This trajectory is not well explained as cultural variation but as a process of ethical refinement driven by the identification and correction of prior failures. The increasing centrality of autonomy illustrates how ethical norms develop in response to recognized problems, supporting the view that ethical change in medicine can constitute genuine progress rather than mere variation across contexts.

## 5 Cultural sensitivity without moral relativism: A three-level framework of ethical analysis

Cultural diversity is an undeniable feature of contemporary medical practice. Differences in values, beliefs, and social structures shape how patients and clinicians understand illness and engage with healthcare. Sensitivity to these differences is ethically required, as it supports communication, trust, and patient-centered care. However, cultural sensitivity must be clearly distinguished from moral relativism: respect for cultural diversity does not entail that ethical obligations vary according to the patient's cultural background.

A first clarification concerns the asymmetry of ethical obligations in the clinical relationship. Ethical duties in medicine are not identical for all agents involved. Physicians are bound by professional obligations, such as the duty to provide truthful information, to obtain informed consent, and to act in the patient's best interests—whereas patients are not subject to the same normative requirements. As emphasized by Beauchamp and Childress, professional ethics imposes specific obligations on clinicians that do not symmetrically apply to patients (Beauchamp and Childress, 2019).

International ethical frameworks explicitly reject the conflation of cultural sensitivity with moral relativism. The UNESCO Universal Declaration on Bioethics and Human Rights affirms that individual autonomy cannot be replaced by collective or cultural authority and that cultural diversity cannot be invoked to infringe upon human dignity, human rights, or fundamental ethical principles (UNESCO, 2005, Articles 6.3 and 12). These statements establish clear limits to cultural accommodation in clinical practice.

**Fig. 3:**
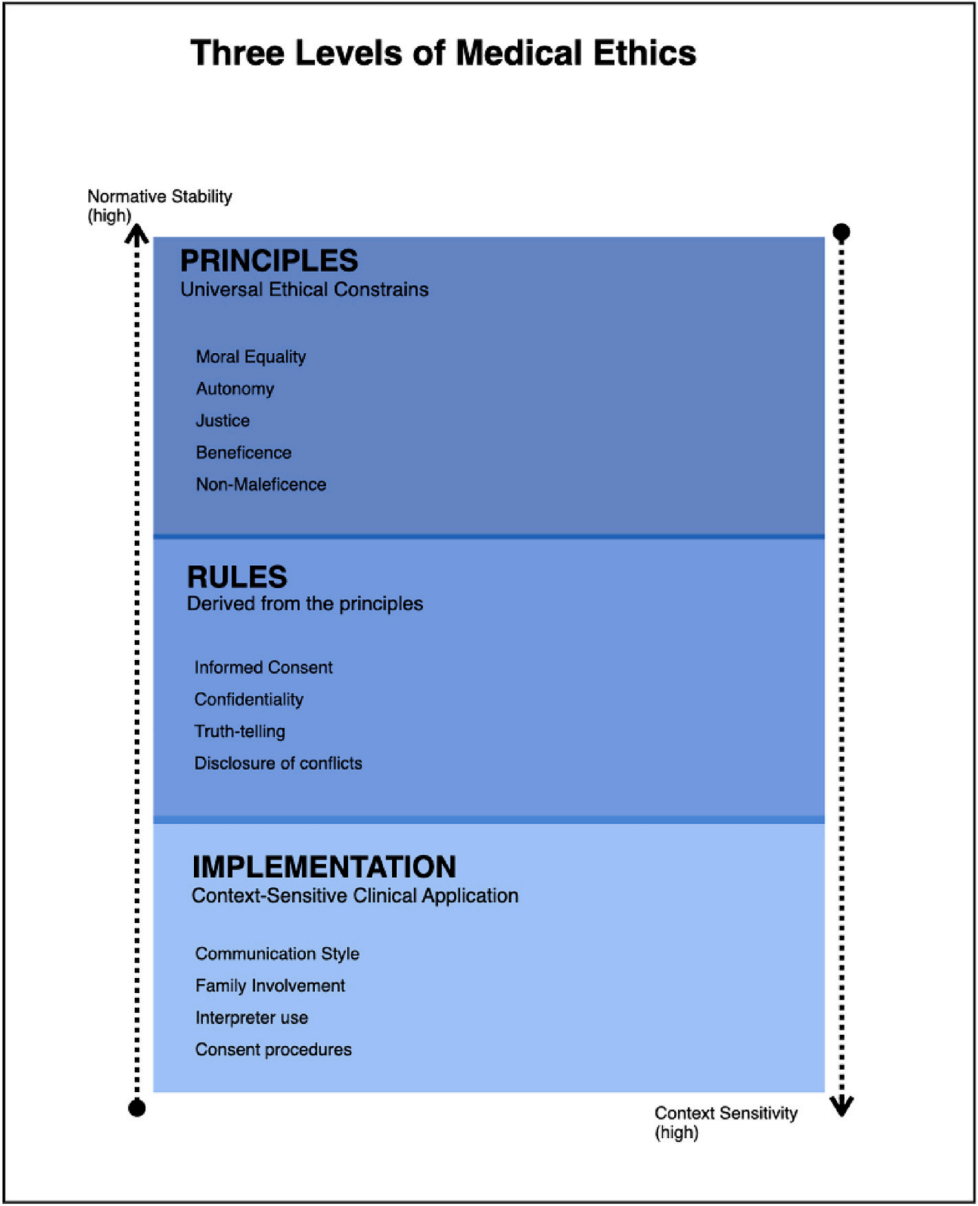
Three levels of medical ethics. Ethical reasoning in medicine operates across three levels that differ in normative stability. Universal principles function as binding constraints that apply equally to all persons. Ethical rules specify these principles into revisable norms governing clinical practice and research. At the level of implementation, these rules are applied in context-sensitive ways that may legitimately vary across cultural and institutional settings. Contextual variation therefore occurs primarily at the level of implementation.

To resolve the tension between universality and cultural diversity, three levels of ethical analysis must be distinguished: (1) universal ethical principles, (2) intermediate rules, and (3) context-dependent clinical practices (Fig. 3). The universal principles of respect for persons and non-maleficence define ethical obligations that apply equally to all patients. Intermediate rules involve the interpretation and specification of these principles in particular clinical situations. Clinical practices concern the concrete implementation of these judgments in context, including culturally sensitive communication, family involvement, and decision-making processes.

Cultural sensitivity properly belongs to the level of implementation: it guides how ethical obligations are fulfilled, not whether they apply. Variations in communication, decision-making processes, or family involvement may be appropriate and even necessary in different cultural contexts, but they do not justify altering underlying ethical standards. To treat cultural norms as determinants of ethical validity would be to conflate descriptive practices with normative claims, committing the is–ought error. An ethically coherent approach to medicine therefore combines universal ethical obligations with context-sensitive practice: all patients are entitled to the same fundamental protections, while clinicians remain attentive to cultural differences in how care is delivered.

## 6 Implications for clinical practice and ethics committees

Clinical decision-making in contemporary medicine increasingly occurs in multicultural settings, where differences in values, expectations, and social norms may complicate ethical deliberation. The challenge is not merely to accommodate diversity, but to do so without compromising the universality of ethical obligations. This requires a clear distinction between universal principles and context-sensitive implementation: while communication strategies and decision-making processes may be adapted to cultural contexts, the underlying ethical standards guiding clinical care must remain constant (Beauchamp and Childress, 2019; UNESCO, 2005). This challenge can be addressed by distinguishing three levels of clinical ethics (Fig. 3) and by approaching ethical dilemmas as problems to be critically examined rather than accommodated or avoided.

Critical Rational Bioethics offers a framework for addressing these challenges by shifting the focus of ethical reasoning from the justification of existing judgments to the resolution of concrete ethical problems. While problem-oriented approaches in clinical ethics, including pragmatist models, also aim to resolve practical dilemmas, Critical Rational Bioethics differs in that it explicitly requires the use and continuous critical refinement of the best available ethical knowledge, including ethical principles, philosophical analysis, scientific evidence, and structured levels of ethical analysis. Rather than relying on the intuitions of supposedly competent or unbiased judges, it assumes fallible agents and subjects their conjectures to critical scrutiny (Popper, 1963; Deutsch, 2011). In clinical practice, this approach encourages physicians to identify dilemmas explicitly, formulate tentative courses of action as conjectures, and test them against potential harms, inconsistencies, and unintended consequences. This supports a dynamic and self-corrective form of ethical reasoning suited to complex and evolving clinical environments.

The implications are equally significant for ethics committees and guideline development. Committees are often tasked with producing recommendations that are both consistent and broadly applicable. Within coherence-based frameworks such as reflective equilibrium, this may lead to harmonizing positions in ways that obscure underlying tensions or unresolved problems (Rawls, 1999; Beauchamp and Childress, 2019). A problem-driven approach, by contrast, encourages committees to foreground ethical dilemmas, explore competing solutions, and critically evaluate their consequences. Guidelines are thus understood as provisional responses but are evaluated through the critical identification of errors rather than through the achievement of coherence among existing positions.

Adopting such an approach may help prevent ethical regression. Historically, problematic practices have often been sustained by unchallenged assumptions, professional authority, or appeals to prevailing norms (Annas and Grodin, 1992; The Belmont Report, 1979). Critical Rational Bioethics addresses this risk by requiring that ethical decisions be both informed by the best available ethical knowledge and subjected to ongoing critical scrutiny. A critical and anti-dogmatic stance ensures that ethical claims remain open to questioning, particularly in contexts where cultural norms, institutional pressures, or scientific interests may influence clinical judgment. Ethical progress, and the avoidance of regression, thus depends on the systematic identification of error and the willingness to revise established practices accordingly (Popper, 1963; Deutsch, 2011).

Ethical reasoning in medicine should therefore be understood as an ongoing and open-ended endeavor. No ethical framework provides final answers, and no set of principles is beyond revision. By adopting a problem-driven, critical, and fallibilist approach, clinicians and institutions can respond more effectively to the complexity of contemporary practice and emerging technological challenges while preserving the universal ethical commitments that underpin medicine (Stengel et al., 2024; 2025; Ganau, 2014; Khilar et al., 2025; Samprón et al., 2025). Ethical progress, on this view, arises not from the stabilization of coherent systems alone, but from the continuous identification and correction of error.

## 7 Discussion

### 7.1 Ethical progress, regression, and the limits of relativism

Contemporary medical ethics operates under a dual demand: the need for stable and binding ethical obligations in clinical practice, and the need for ongoing critical reflection in response to evolving scientific, technological, and social contexts. The World Medical Association's International Code of Medical Ethics emphasizes the obligation to uphold consistent ethical standards, while the UNESCO Universal Declaration on Bioethics and Human Rights highlights the necessity of articulating universal principles capable of guiding responses to increasingly complex dilemmas. Together, these demands of ethical stability and continuous revision, define the central tension that frames ethical reasoning in medicine.

Medical ethics is not a static body of norms but a dynamic field capable of change and improvement. Historical developments show that major advances in bioethics have often followed the recognition of ethical failures, prompting critical reassessment and reform (Baker, 1998a, 1998b). However, the capacity for progress does not preclude the possibility of stagnation or regression. Ethical standards may deteriorate, and previously abandoned forms of practice, typically strong paternalism or the neglect of patient autonomy, may re-emerge under new rationalizations. This pattern suggests that ethical development is best understood not as the accumulation or stabilization of prevailing beliefs, but as a process driven by the identification of error and its correction.

A central risk in this context is moral relativism. If ethical standards are treated as dependent on cultural or social context without reference to universal normative constraints, then harmful practices may be sustained under the guise of local norms. Historical and contemporary examples illustrate this danger, including unethical human experimentation, the withholding of effective treatment from vulnerable populations, and the defense of harmful practices through appeals to cultural or ideological frameworks (Annas and Grodin, 1992; Leaning, 1996; Young et al., 2020). In each case, ethical failure was accompanied by forms of reasoning that subordinated universal obligations to contextual norms. These episodes also reveal that progress followed from criticism and reform, not from the acceptance of prevailing beliefs. This raises a central question: how should ethical knowledge in medicine be generated and improved? The present work addresses this question by proposing Critical Rational Bioethics as a problem-driven, fallibilist alternative to coherence-based approaches.

### 7.2 Foundations of ethical knowledge: Objectivity and moral equality

Appeals to “common morality” or to the judgments of “morally committed persons,” as described by Beauchamp and Childress, are often invoked to support the existence of a shared moral framework across cultures (Beauchamp and Childress, 2019). However, this approach faces a fundamental difficulty: descriptive accounts of what people believe or accept cannot, by themselves, ground normative claims about what ought to be required. As Hume's is–ought distinction makes clear, deriving ethical norms from prevailing moral beliefs risks conflating sociological description with normative evaluation (Savulescu, 2015). In medicine, this problem is reinforced by empirical evidence showing substantial variation in moral beliefs across societies and over time, challenging the assumption of a stable and universally shared common morality (Inglehart et al., 2014; Karlsen and Solbakk, 2011; Beck, 2015). Ethical knowledge therefore cannot be reduced to the expression of collective preferences or the aggregation of widely held views. Rather, it has an objective dimension: it concerns better or worse ways of acting in relation to harm, injustice, and human well-being, and must be assessed through critical scrutiny rather than conformity to prevailing beliefs.

Moral equality constitutes a central normative commitment of medical ethics. It affirms that all human beings possess the same moral worth and are therefore entitled to equal ethical consideration, irrespective of culture, social status, or personal characteristics. In clinical practice, this principle functions as a binding constraint: patients cannot be subject to different ethical standards on the basis of cultural identity without undermining the moral legitimacy of medicine itself. Yet its practical authority should not be confused with dogmatic finality. In a fallibilist framework, moral equality remains open to critical scrutiny in the sense that its scope and implications—such as questions concerning moral status in contexts like brain death, organ donation, or early human development—may require further clarification and refinement. Such scrutiny, however, cannot justify diminishing equal moral consideration or reintroducing differential standards of care. Moral equality therefore has a dual role: it provides stability in practice while remaining open to epistemic refinement. This clarifies a central point of the present argument: cultural sensitivity may shape how ethical obligations are implemented, but not whether equal ethical obligations apply.

### 7.3 Universalism, critiques, and implications

Critical Rational Bioethics has important implications for both clinical practice and ethics committees. In place of approaches centered on the application of pre-existing norms or the harmonization of intuitions, it frames ethical deliberation as the critical examination of concrete problems. In clinical settings, clinicians are encouraged to identify dilemmas explicitly, formulate provisional responses informed by the best available ethical knowledge in terms of ethical principles, relevant scientific evidence, and the structured differentiation of levels of ethical analysis. Next this provisional judgment should be tested them against hypothetical potential harms, inconsistencies, and unintended consequences. For ethics committees, emphasis is shifted from producing consensus, at the frequent cost of leaving underlying conflicts insufficiently examined—to exposing unresolved problems, comparing competing solutions, and revising recommendations in light of criticism. In multicultural contexts, this is especially important, as it allows cultural sensitivity in communication and implementation while preserving universal ethical obligations toward all patients.

The distinction between universal ethical obligations and their context-dependent implementation becomes particularly relevant in resource-limited healthcare systems. Differences in material resources, infrastructure, legal frameworks, or cultural traditions undoubtedly influence the clinical options available to physicians. However, these factual constraints do not imply different ethical standards for different societies. Rather, they require clinicians to determine, through critical evaluation, how universal ethical obligations can best be fulfilled under existing circumstances. Consequently, Critical Rational Bioethics distinguishes between ethical universality at the level of principles and legitimate variation at the level of implementation. This distinction applies equally to multicultural societies, humanitarian settings, and healthcare systems with limited resources.

Beyond its practical implications, Critical Rational Bioethics offers a distinct theoretical advantage by providing an account of how ethical knowledge can develop and improve. Rather than grounding ethical claims in coherence among existing beliefs or in widely shared norms, it treats them as conjectural solutions to problems, to be critically assessed and revised in light of their consequences. This allows ethical reasoning to incorporate and progressively refine the best available ethical knowledge, including principles, philosophical analysis, and scientific understanding, while remaining open to correction. In this way, ethical progress is understood not as the stabilization of agreement, but as the systematic identification and elimination of error, enabling the development of better, albeit provisional, solutions to ethical problems.

At the same time, this approach has limitations. The present analysis is primarily conceptual, and further work is required to operationalize Critical Rational Bioethics in specific clinical and institutional contexts. Questions remain regarding how best to structure processes of criticism, how to evaluate competing moral judgments in practice, and how to ensure that such scrutiny enables the critical identification and correction of error. Moreover, while the framework emphasizes the identification and elimination of error, determining what constitutes a better ethical solution may itself be contested and require ongoing clarification. These limitations, however, are consistent with its fallibilist commitments: ethical knowledge remains provisional and open to revision. In this sense, Critical Rational Bioethics does not offer final answers, but a method for their continuous improvement, reinforcing the view of medicine as an evolving ethical practice grounded in critical inquiry and the reduction of harm.

## 8 Conclusions

This work has argued for the reaffirmation of moral equality as a central normative commitment of medical ethics. All patients, irrespective of cultural, social, or contextual differences, must be regarded as possessing equal moral worth and therefore entitled to the same fundamental ethical protections. On this basis, cultural relativism cannot provide a viable framework for medical ethics: by allowing ethical obligations to vary across contexts without universal constraints, it undermines moral equality and fails to distinguish between ethically sound practices and harmful ones. Respect for cultural diversity remains essential, but it cannot justify departures from fundamental ethical obligations without compromising the integrity of medicine as a moral practice.

The analysis further supports an account of ethical progress in medicine grounded in the identification and correction of error. Historical developments in bioethics show that advances have typically followed the recognition of harmful or inadequate practices, leading to their critical reassessment and reform. The emergence and consolidation of principles such as respect for autonomy reflect not cultural preference, but improvements in ethical understanding aimed at preventing harm, coercion, and injustice. Ethical knowledge, on this view, is objective yet fallible: it does not derive from consensus or tradition, but from its capacity to withstand critical scrutiny and to reduce avoidable harm.

Within this perspective, ethical universalism and cultural sensitivity are not opposing positions but can be coherently reconciled. Universal ethical principles define the obligations that apply equally to all patients, while cultural sensitivity informs how these obligations are communicated and implemented in practice. Critical Rational Bioethics provides a framework for this integration by treating ethical reasoning as a problem-driven and fallibilist process in which moral judgments are continuously subjected to critical scrutiny and improvement. In this sense, medical ethics is best understood not as a fixed system of norms, but as an evolving practice committed to the refinement of ethical knowledge through the identification and elimination of error, in response to shared human vulnerabilities.

## Declaration of the use of generative artificial intelligence in this paper

During the preparation of this work, the authors used generative artificial intelligence (ChatGPT, OpenAI) to assist with language refinement, clarity, and structure of the manuscript. The authors reviewed and edited all content and take full responsibility for the final version of the manuscript.

## Declaration of competing interest

The authors declare that they have no known competing financial interests or personal relationships that could have appeared to influence the work reported in this paper.
